# Potentiometric detection and removal of copper using porphyrins

**DOI:** 10.1186/1752-153X-7-111

**Published:** 2013-07-06

**Authors:** Dana Vlascici, Iuliana Popa, Vlad A Chiriac, Gheorghe Fagadar-Cosma, Horia Popovici, Eugenia Fagadar-Cosma

**Affiliations:** 1 West University of Timisoara, Faculty of Chemistry-Biology-Geography, Pestalozzi Street 16, Timisoara, 300115, Romania; 2 National Institute of Research for Electrochemistry and Condensed Matter, Timisoara, Aurel Paunescu Podeanu Street 144, Timisoara, 300860, Romania; 3 "Politehnica" University of Timisoara, 2 T. Lalescu Street, Timisoara, 300223, Romania; 4 Institute of Chemistry Timisoara of Romanian Academy, M. Viteazul Ave. 24, Timisoara, 300223, Romania

**Keywords:** Porphyrins, Ion-selective electrode, Potentiometry, Copper, PVC membrane, Detection, Removal

## Abstract

**Background:**

Copper is an essential trace element with a great importance in industry, environment and biological systems. The great advantage of ion-selective sensors in comparison with other proposed techniques is that they are measuring the free metal ion activity which is responsible for their toxicity. Porphyrins are known to be among the best ionophores in formulation of ion-selective sensors.

**Results:**

A symmetrically substituted *meso*-porphyrin, namely: 5,10,15,20-tetrakis(4-allyloxyphenyl)porphyrin (TAPP) was used in the construction of a new copper selective-sensor and was also tested for the removal of copper from waste waters. The potentiometric response characteristics (slope and selectivity) of copper-selective electrodes based on TAPP in *o*-nitrophenyloctylether (*o*-NPOE), dioctyl phtalate (DOP) and dioctyl sebacate (DOS) plasticized with poly(vinyl chloride) membranes are compared.

**Conclusions:**

The best results were obtained for the membrane plasticized with DOP. The sensor has linear response in the range 1x10^-7^ – 1x10^-1^ M with 28.4 ± 0.4 mV/decade near-Nernstian slope towards copper ions and presents good selectivity. Due to its chelating nature, the same porphyrin was also tested for the retention of copper from synthetic copper samples, showing a maximum adsorption capacity of 280 mg/g.

## Background

Trace metals are toxic for many life forms when their concentration exceeds a certain limit. This is the reason why their presence in the environment is an important problem and must be precisely monitored. Copper is an essential trace element with a great importance in industry, environment and biological systems. Considered to be the second toxic metal to aquatic life, copper appears in waters and wastewaters from mining industries, refineries, paper and dyeing. Besides, in the recycling process of Li-batteries technical developments regarding Ni, Co and Mn recovery implies copper monitoring in synthetic leach liquor resulted from reductive leaching. Due to the increased interest in environmental protection, both its detection and removal are very important and many methods were used during the time for this purpose.

Several techniques were used for copper monitoring such as: atomic absorption spectrometry (AAS), UV–Vis spectrometry and inductively coupled plasma atomic emission spectrometry (ICP-AES), high performance liquid chromatography (HPLC), anodic stripping voltammetry, cyclic voltammetry [1-5]. Generally, these methods requires expensive instruments, qualified personnel, sample pretreatment and are hard to use in environmental conditions.

The potentiometric method with ion-selective sensors was widely developed in the last years due to its simplicity, low cost and fast analysis and a lot of the reported sensors were used in the environmental analysis [6]. The great advantage of ion-selective sensors is that they are measuring the free metal ion activity which is responsible for their toxicity. This is the reason why a lot of copper-selective sensors based on different ionophores were reported. Several organic compounds, such as: 1-(2-hydroxybenzylidene) thiosemicarbazide [7], Schiff bases [8,9], 2-mercaptobenzoxazole [10], bezo-substituted macrocyclic diamide [11], 6-methyl-4-(1-phenylmethylidene)amino-3-thioxo-1,2,4-triazin-5-one [12], porphyrin derivatives [13], cyclic tetrapeptide derivatives [14], 7-hydroxy-3-(2-methyl-2,3-dihydrobenzo[d]thiazol-2-yl)-2H-chromen-2-one [15], tetraazacyclotetradecane derivative [16], dimethyl 4,4’-(*o*-phenylene)bis(3-thioallophanate) [17], succinimide derivative [18], polyindole [19] were tested as ionophores.

For copper removal, many materials and waste materials were reported in the literature [20-26]. The efficiency of removing the metals from wastewaters by adsorption method depends on the physical and chemical composition of the adsorbents.

**Scheme 1:**
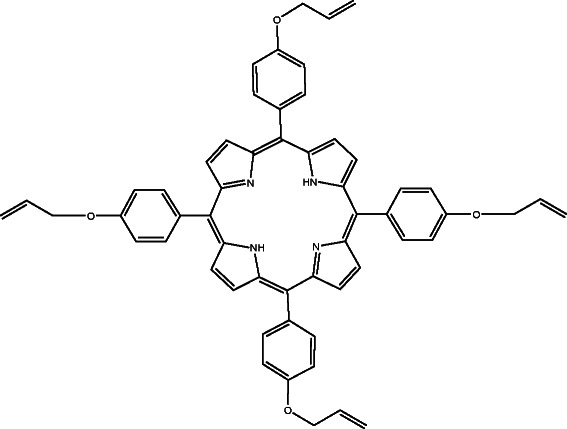
**The structure of the TAPP porphyrin.** The figure presents the chemical structure of 5,10,15,20-tetrakis(4-allyloxyphenyl) porphyrin (TAPP) which was used in the paper as ionophore in a new copper-selective sensor and as adsorbent for copper removal from synthetic samples.

In the present paper, 5,10,15,20-tetrakis(4-allyloxyphenyl)porphyrin (TAPP) (Scheme 1) was used in the formulation of a new copper-selective sensor and also tested for the removal of copper from synthetic waste waters. The best obtained sensor has a linear response in the in the range 1x10^-7^ – 1x10^-1^ M with near-Nernstian slope of (28.4 ± 0.4) mV/decade towards copper ions, in a pH range from 2 to 8, with a detection limit of 9x10^-8^ M. The removal tests were conducted using 5 mg of the same TAPP porphyrin and two copper synthetic samples of 20 and 50 mg/L concentrations. The maximum adsorption capacity was of 280 mg/g.

## Results and discussion

### Detection tests

#### Working concentration range and slope

The behaviour of any ion-selective sensor depends on the nature and structure of the ionophore used in the membrane composition. Due to the fact that free porphyrins have donor atoms in their structure, the expectations are to have good affinity to transition metals ions. This was proved by several papers reported in the literature [13,27,28] that mention different porphyrins as sensing material. Besides the critical role of the ionophore, the nature of the plasticizers having different dielectric constants influence the mobility of the ionophore in the membrane phase. The selection of the best plasticizer can improve the sensor potentiometric answer in terms of sensitivity and sometimes also of selectivity. There are some reports which recommend lower dielectric constant plasticizers for some divalent metal selective sensors [14] and others in which the best results were obtained by using plasticizers characterized by big dielectric constants [18]. In this respect, this work was focused on obtaining and comparatively presentation of three sensors having the same weight percentage composition of the membrane, but using three different plasticizers: sensor A – plasticized with *o*-nitrophenyloctylether (*o*-NPOE, Ɛ = 24), sensor B – plasticized with dioctyl phtalate (DOP, Ɛ = 7) and sensor C – plasticized with dioctyl sebacate (DOS, Ɛ = 4).

**Figure 1:**
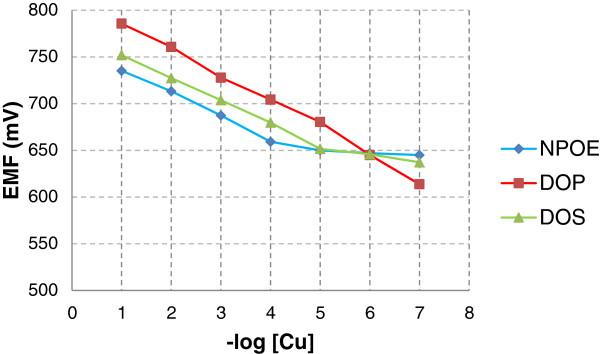
**The potentiometric copper responses of electrodes A-C.** The figure presents the potentiometric responses of the new obtained sensors based on TAPP plasticized with A – NPOE, B – DOP and C – DOS.

The three sensors were tested in copper solutions from 10^-7^ – 10^-1^ M and the obtained results are presented in Figure 1 and Table 1. Analyzing the results, it seems that all of them are copper-selective, having near-Nernstian slopes in different concentration ranges. Sensor B, plasticized with DOP works in the widest concentration range from 1x10^-7^ – 1x10^-1^ M, with a slope of (28.4 ± 0.4) mV/decade.

**Table 1:** Potentiometric response characteristics of the copper-selective sensors A-C

**Sensor**	**Working conc. range (M)**	**Slope (mV/decade)**	**Detection limit (μM)**	**R**^**2**^
A	5x10^-5^ – 1x10^-1^	(26.0 ± 0.3)	0.8	0,9969
B	1x10^-7^ – 1x10^-1^	(28.4 ± 0.4)	0.09	0,9972
C	1x10^-5^ – 1x10^-1^	(24.8 ± 0.4)	8.0	0,9989

The table presents the working concentration range (M), slope (mV/decade of activity) and detection limit (μM) of the obtained sensors.

From the obtained data it can be highlighted that there is no specific rule in choosing the plasticizer due to the fact that DOP has medium values of the dielectric constant comparatively with those of NPOE and DOS, so that selecting of one plasticizer should be made after performing tests.

#### Effect of interfering ions

The selectivity coefficients show the influence of the interfering ions on the potential response of the sensor. They were determined by separate solution method using equation (1) and describe the preference of the sensor for the primary ion (Cu^2+^) relative to the interfering ions, which in our case were: monovalent (Na^+^, Li^+^) , divalent (Ni^2+^, Mn^2+^, Co^2+^, Zn^2+^, Pb^2+)^ and trivalent (Fe^3+^ and Al^3+^). The obtained values are presented in Table 2.

**Table 2:** Selectivity coefficients of the obtained sensors

**Interfering ion (X)**	**Electrode A**	**Electrode B**	**Electrode C**
Ni^2+^	- 4.38	- 5.56	- 5.84
Mn^2+^	- 1.60	- 3.28	- 1.03
Co^2+^	- 3.67	- 4.16	- 2.99
Fe^3+^	+ 1.27	- 0.80	- 0.92
Na^+^	- 1.70	- 1.68	- 1.87
Li^+^	- 2.33	- 3.74	- 2.08
Zn^2+^	- 1.00	- 2.27	−3.19
Pb^2+^	-	−1.26	-
Al^3+^	-	−2.73	-

The table presents the selectivity coefficients of the obtained sensors calculated using equation (1) towards a number of 9 cations: Ni^2+^, Mn^2+^, Co^2+^ Pb^2+^, Zn^2+^, Fe^3+^, Al^3+^, Na^+^ and Li^+^.

The data presented in Table 2 put into evidence that also in the terms of selectivity the best results were obtained for sensor B, having the membrane plasticized with DOP. The sensor has very good selectivity in comparison with the other tested cations and was further used in all the determinations.

From Table 2, it results that the sensor A has Fe^3+^ as interfering ion, but it could not be declared as an iron-selective sensor due to the values of the slopes which are sub-Nernstian.

#### Effect of pH

**Figure 2:**
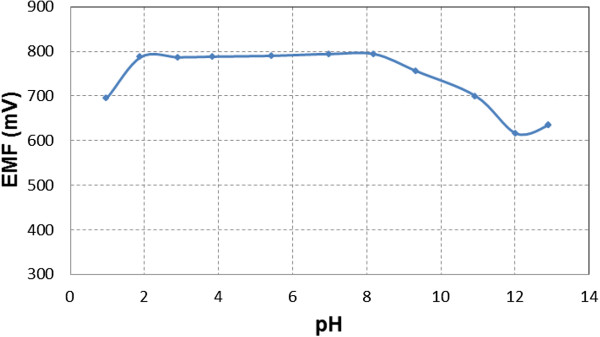
**The pH effect of the test solution on the potential response of the best obtained sensor.** The figure presents the influence of pH on the potential response of the sensor. Solutions having different values of the pH were used.

The pH function of sensor B was obtained in solutions having different values of the pH and is presented in Figure 2. The potential of the sensor determined as a function of pH remains constant over the pH range from 2 to 8, which may be taken as the working pH range of the sensor.

#### Response time and lifetime

The average time for the copper-selective electrode to reach 95% of the final potential value after successive immersion of the electrode in a series of copper ion solutions, each having a 10-fold difference in concentration, was measured. The response time from 10^-3^ to 10^-2^ M copper solutions was of 10 s, but it became longer for diluted solutions.

One of the most important characteristics of a sensor is its lifetime. In our case, the sensor having the membrane plasticized with DOP has also the best lifetime, of 6 weeks. During this period of time no significant change of the potential was noticed. The stability and reproducibility of the best obtained sensor were also tested. The standard deviation of 15 replicate measurements made for the 1 × 10^−3^ M solution was ±0.4 mV.

The potentiometric response characteristics of the present sensor comparatively to those of some other sensors reported in the literature are presented in Table 3.

**Table 3:** Response characteristics of the proposed sensor comparatively to other similar electrodes presented in the literature

**Ref.**	**Linear range**	**Slope (mV/decade)**	**Response time (s)**	**Detection limit (M)**
	**(M)**			
[7]	1x10^-5^ – 1x10^-1^	28.6 ± 0.4	25	5.4x10^-6^
[8]	1x10^-6^ – 1x10^-1^	29.8 ± 0.7	15	6.0x10^-7^
[9]	1x10^-8^ – 5.7x10^-4^	-	150	8.8x10^-9^
[10]	5x10^-6^ – 1.6x10^-2^	29.2 ± 2.0	<10	2x10^-6^
[11]	1x10^-7^ – 1x10^-1^	27.9 ± 0.8	<30	7,8x10^-8^
[12]	1x10^-6^ – 1x10^-1^	29.2 ± 0.4	-	30,5 μg/L
[13]	4.4x10^-6^ – 1x10^-1^	29.3	8	0.28 mg/L
[14]	1x10^-6^ – 1x10^-2^	30.2 – 25.9	<15	0.05-0.13 mg/L
[15]	1x10^-6^ – 1x10^-1^	29.6 ± 0.3	13	7.9x10^-7^
[16]	1x10^-6^ – 1x10^-1^	28.8	10-40	7x10^-7^
[17]	9.8x10^-6^ – 1x10^-1^	30.3	20	-
[18]	1x10^-5^ – 1x10^-2^	37.6	0.25	4.4x10^-6^
This work	1x10^-7^ – 1x10^-1^	28.4 ± 0.4	10	9x10^-8^

#### Analytical applications

### Determination of copper from synthetic solutions

For analytical application, the sensor was tested for copper-detection in two synthetic samples, comparatively to the AAS method. The samples of 20 and 50 mg/L copper were further used for the removal tests using as adsorbent the same TAPP porphyrin. The results are presented in Table 4 for five replicates and they were found to be in close agreement.

**Table 4:** Determination of copper in synthetic samples

**Copper sample (mg/L)**	**Found by electrode (mg/L)**	**Found by AAS (mg/L)**	**Recovery (%)**
20	(19.3 ± 0.4)	(19.6 ± 0.2)	98.4
50	(49.5 ± 0.5)	(49.8 ± 0.3)	99.4

The table presents the application of the best obtained sensor in detection of copper from two different synthetic samples of 20 and 50 mg/L copper concentration, comparatively with those obtained by AAS.

#### Determination of copper from spent lithium ion batteries

The synthetic leach liquor composition was: 50 g/L of Co, 10 g/L of Li, 7 g/L of Al, 5 g/L of Ni, 2 g/L of Fe, 3 g/L of Cu, 1.5 g/L of Mn. Due to the ionic strength of the synthetic solution, sodium nitrate was added to copper samples to brought them to the same ionic strength [29]. The obtained results using the sensor based on three measurements were of 2.97 ± 0.05 g Cu/L.

### Removal tests

**Figure 3:**
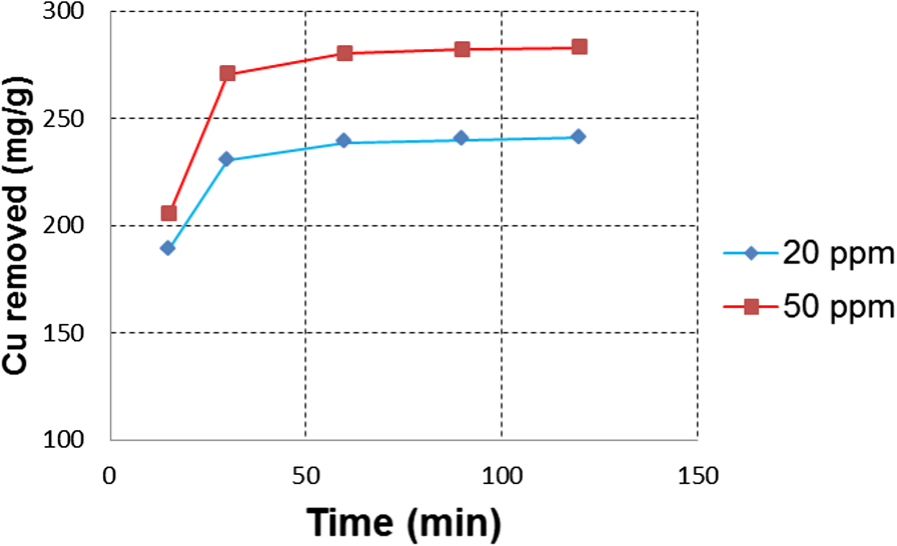
**Adsorption isotherms of copper(II) with different initial concentrations on TAPP.** The figure presents the adsorption rate for two different initial copper concentrations of 20 and 50 mg/L, at different time intervals.

Due to the fact that 5,10,15,20-tetrakis(4-allyloxyphenyl)porphyrin was found to be a good ionophore for copper(II) ions, we have tried to use the same TAPP porphyrin as copper adsorbent material. For this purpose, the two synthetic samples used in the analytical application of the sensor, of 20 and 50 mg/L Cu^2+^ were further used for the adsorption experiment. The adsorption rate has been analysed using equation (2) and plotted in Figure 3.

The copper concentration was increased from 20 to 50 mg/L to obtain the maximum removal capacities for the targeted copper ions. High removal rates were obtained at the beginning of the experiment and the equilibrium was attained in about 60 min. After that time, a plateau was established. The maximum adsorption was of 280.2 mg of copper per gram of adsorbent (TAPP). The distribution coefficients, which show the affinity of the adsorbent towards the copper ions, were calculated according to equation (3) and the removal capacities, according to equation (4). All the values are listed in Table 5.

**Table 5:** Copper adsorption capacity, distribution coefficient and removal capacity

**C**_**0 **_**(mg/L)**	**Q**_**e**_**(mg/g)**	**K**_**D**_	**% Removal**
20	238,8	29,6	59,7
50	280,2	7,8	28,0

The table presents the application of 5,10,15,20-tetrakis(4-allyloxyphenyl)porphyrin as adsorbent for copper removal from two different copper solutions in terms of Q_e_, K_D_ and% Removal calculated using equations (2), (3) and (4).

Analysing the values, it results that TAPP can also be used with good results as an efficient adsorbent for copper(II) ions.

## Conclusions

The detection and removal of copper ions using 5,10,15,20-tetrakis(4-allyloxyphenyl)porphyrin (TAPP) was investigated. Porphyrin (TAPP) was embedded as ionophore in a PVC matrix, using dioctyl phtalate (DOP) as plasticizer, for obtaining a new copper-selective sensor. The resulted sensor is characterized by good sensitivity, very good selectivity and short response time of 10 s. The electrode was used for the potentiometric detection of copper in synthetic samples with a good precision level. The same TAPP porphyrin was tested for the retention of copper from synthetic copper samples with a maximum adsorption capacity of 280 mg/g.

## Methods

### Reagents

The porphyrin 5,10,15,20-tetrakis(4-allyloxyphenyl)porphyrin (TAPP) was synthesized, purified and characterized in accordance with previously published procedures [30]. For membrane preparation, poly(vinyl)chloride (PVC) high molecular weight, bis(2-ethylhexyl)sebacate (DOS), *o*-nitrophenyloctylether (NPOE), dioctylphtalate (DOP), sodium tetraphenylborate (NaTPB) and tetrahydrofurane (THF) were purchased from Fluka and Merck. All salts, acids and base were of analytical reagent grade. Double distilled water was used. The performance of each sensor was investigated by measuring its potential in the concentration range 10^-5^- 10^-1^ M of different cationic solutions. In the case of copper(II) the solutions were made in a concentration range up to 10^-8^ M. Stock solutions, 0.1 M, were prepared by dissolving metal nitrates in double distilled water and standardized if necessary. All working solutions were prepared by gradual dilution of the stock solutions.

### Electrode membrane preparation and measurements

The membranes have the weight percentage composition as follows: 1% (0,005 g) ionophore, 33% (0,165 g) PVC and 66% (0,330 g) plasticizer. Sodium tetraphenylborate was used as additive (20 mol% relative to ionophore). The electroactive material and the solvent mediator were mixed together, and then the PVC and the appropriate amount of THF (3–5 mL) were added and mixed to obtain a transparent solution. This solution was transferred onto a glass plate of 20 cm^2^, and the THF was allowed to evaporate at room temperature leaving a tough, flexible membrane embedded in a PVC matrix. The round shape pieces of membranes (diameter = 8 mm) were cut out and assembled on the Fluka electrode body. The measurements were carried out at room temperature using a Hanna Instruments HI223 pH/mV-meter by setting up the following cell:

Ag|AgCl|KNO_3_ (0.1 M)|sample|ion-selective membrane|0.01 M KCl|AgCl, Ag.

Prior to EMF measurements, all the sensors were conditioned for 24 h by soaking in 0.01 M Cu^2+^ solution. Potentiometric selectivity coefficients were determined according to the separate solution method [31] using the experimental EMF values obtained for 0.01 M of the tested cations and a theoretical slope of 29.6 mV/decade of activity for copper(II) cation, calculated by the equation (1):

(1) $$\log K_{X,Y}^{\mathrm{pot}}=\frac{\left(E_{Y}-E_{x}\right)\cdot z_{X}\cdot F}{\mathrm{RT}\ln10}+\left(1-\frac{z_{X}}{z_{Y}}\right)\mathrm{lg}a_{X}$$

The detection limit of each sensor was established at the point of intersection of the extrapolated linear mid-range and final low concentration level segments of the calibration plot. The effect of pH on the potentiometric response of the sensor was obtained by introducing the best obtained sensor in solutions of HNO_3_, NaOH and different buffers, in a pH range from 1 to 12.92.

### Adsorption test

The copper removal experiments were carried out by stirring 5 mg of TAPP in 100 mL of copper solution at 25°C. Two different copper concentrations of 20 and 50 mg/L were tested. The solution from each vial was analysed to determine copper concentration after different time intervals. The removal rate was calculated using equation (2):

(2) $$Q_{e}=\frac{\left(C_{0}-C_{e}\right)\times V}{m}\mathrm{mg}/g.$$

Where Q_e_ is the quantity of copper adsorbed on the porphyrin at the time if equilibrium (mg/g), C_0_ is the initial concentration of copper in the aqueous solution (ppm), C_e_ is the final cooper concentration at the time of equilibrium (ppm), m is the mass of porphyrin used as adsorbent (g), V is the volume of the solution (L).

The distribution coefficient (K_d_) shows the affinity of the adsorbent towards copper ions and was calculated using the equation (3)

(3) $$K_{D}=\frac{Q_{e}}{C_{e}}\left(L/g\right)\;.$$

The removal capacities of copper ions (%) taking into consideration the two different initial concentrations, are calculated by equation (4):

(4) $$\mathrm{Removal}\;\mathrm{capacity}\left(\%\right)=\frac{\left(C_{o}-C_{e}\right)\;}{C_{o}}\times 100.$$

## Competing interests

The authors declare that they have no competing interest.

## Authors’ contributions

DV, IP have made the potentiometric measurements and design the whole concept of research, DV, VAC have made the removal measurements and the initial draft of the manuscript, HP has made the AAS measurements, GFC and EFC have synthesized and characterized the porphyrin ionophore and prepared the final draft of the manuscript. All authors read and approved the final manuscript.
